# Gut Microbiota Alterations and Circulating Imidazole Propionate Levels Are Associated With Obstructive Coronary Artery Disease in People With HIV

**DOI:** 10.1093/infdis/jiad604

**Published:** 2024-01-09

**Authors:** Marius Trøseid, Antonio Molinaro, Marco Gelpi, Beate Vestad, Klaus Fuglsang Kofoed, Andreas Fuchs, Lars Køber, Kristian Holm, Thomas Benfield, Per M Ueland, Johannes R Hov, Susanne Dam Nielsen, Andreas Dehlbæk Knudsen

**Affiliations:** Research Institute of Internal Medicine, Oslo University Hospital Rikshospitalet, Oslo, Norway; Section for Clinical Immunology and Infectious Diseases, Oslo University Hospital Rikshospitalet, Oslo, Norway; Institute of Clinical Medicine, University of Oslo, Oslo, Norway; Research Institute of Internal Medicine, Oslo University Hospital Rikshospitalet, Oslo, Norway; Norwegian PSC Research Center, Department of Transplantation Medicine, Oslo University Hospital, Oslo, Norway; Department of Infectious Diseases, Rigshospitalet, University of Copenhagen, Copenhagen, Denmark; Research Institute of Internal Medicine, Oslo University Hospital Rikshospitalet, Oslo, Norway; Department of Cardiology, Rigshospitalet, University of Copenhagen, Copenhagen, Denmark; Department of Radiology, Rigshospitalet, University of Copenhagen, Copenhagen, Denmark; Department of Cardiology, Rigshospitalet, University of Copenhagen, Copenhagen, Denmark; Department of Cardiology, Rigshospitalet, University of Copenhagen, Copenhagen, Denmark; Research Institute of Internal Medicine, Oslo University Hospital Rikshospitalet, Oslo, Norway; Norwegian PSC Research Center, Department of Transplantation Medicine, Oslo University Hospital, Oslo, Norway; Institute of Clinical Medicine, University of Oslo, Oslo, Norway; Department of Infectious Diseases, Copenhagen University Hospital—Amager and Hvidovre, Hvidovre, Denmark; Bevital, Bergen, Norway; Research Institute of Internal Medicine, Oslo University Hospital Rikshospitalet, Oslo, Norway; Norwegian PSC Research Center, Department of Transplantation Medicine, Oslo University Hospital, Oslo, Norway; Institute of Clinical Medicine, University of Oslo, Oslo, Norway; Section of Gastroenterology, Department of Transplantation Medicine, Oslo University Hospital, Oslo, Norway; Department of Infectious Diseases, Rigshospitalet, University of Copenhagen, Copenhagen, Denmark; Department of Clinical Medicine, University of Copenhagen, Copenhagen, Denmark; Department of Surgical Gastroenterology and Transplantation, Rigshospitalet, University of Copenhagen Copenhagen, Denmark; Department of Infectious Diseases, Rigshospitalet, University of Copenhagen, Copenhagen, Denmark; Department of Cardiology, Rigshospitalet, University of Copenhagen, Copenhagen, Denmark

**Keywords:** gut microbiota, HIV, imidazole propionate, cardiovascular, coronary artery disease

## Abstract

**Background:**

The impact of gut microbiota and its metabolites on coronary artery disease (CAD) in people with human immunodeficiency virus (PWH) is unknown. Emerging evidence suggests that imidazole propionate (ImP), a microbial metabolite, is linked with cardiometabolic diseases.

**Methods:**

Fecal samples from participants of the Copenhagen Comorbidity in HIV infection (COCOMO) study were processed for 16S rRNA sequencing and ImP measured with liquid chromatography-tandem mass spectrometry. CAD severity was investigated by coronary computed tomography-angiography, and participants grouped according to obstructive CAD (n = 60), nonobstructive CAD (n = 80), or no CAD (n = 114).

**Results:**

Participants with obstructive CAD had a gut microbiota with lower diversity and distinct compositional shift, with increased abundance of *Rumiococcus gnavus* and *Veillonella*, known producers of ImP. ImP plasma levels were associated with this dysbiosis, and significantly elevated in participants with obstructive CAD. However, gut dysbiosis but not plasma ImP was independently associated with obstructive CAD after adjustment for traditional and HIV-related risk factors (adjusted odds ratio, 2.7; 95% confidence interval, 1.1–7.2; *P* = .048).

**Conclusions:**

PWH with obstructive CAD displays a distinct gut microbiota profile and increased circulating ImP plasma levels. Future studies should determine whether gut dysbiosis and related metabolites such as ImP are predictive of incident cardiovascular events.

## Teaser

In people with HIV, obstructive coronary artery disease is associated with altered gut microbiota composition and higher levels of imidazole propionate, a microbiota-dependent metabolite.

Despite antiretroviral therapy (ART), people with HIV (PWH) have reduced life expectancy [1], largely due to increased prevalence of noncommunicable diseases, including cardiovascular disease [2]. While the gut microbiota has been suggested to contribute to cardiometabolic disorders in the uninfected population [3], the determinants of this association are unclear. Disease-associated alterations in microbiota composition, disruption of the gut barrier, microbial toxins, and subsequent inflammation have all been associated with the development of cardiometabolic disorders [4].

The potential association between human immunodeficiency virus (HIV) and gut microbiota alterations has been investigated in several studies, with conflicting results [5–10]. A shift from a *Bacteroides*-enriched to a *Prevotella*-enriched phenotype was reported in several studies, but has later been linked to sexual practice, particularly men who have sex with men (MSM) [11]. In subsequent studies controlling for MSM, other microbiota traits have been associated with HIV, in particular increased abundance of proinflammatory proteobacteria and reduced clostridia, including known producers of butyrate, an important substrate for maintaining the gut barrier [12].

We recently reported results from Copenhagen Comorbidity in HIV infection (COCOMO) study [13] suggesting that specific gut microbiota changes, including increase in gammaproteobacteria and reduction in butyrate-producing bacteria accompany HIV infection, also when controlling for MSM status. Microbiota compositional shift in HIV was associated with accumulation of visceral adipose tissue and presence of the metabolic syndrome [14]. However, whether such microbiota alterations and microbial-derived metabolites could be related to coronary artery disease (CAD) is currently unknown.

Microbiota traits vary from individual to individual and are affected by several confounding factors, including sexual practice and medicines, whereas circulating metabolites may be less variable and therefore easier to evaluate as biomarkers. The gut microbiota produces several metabolites that have been linked to cardiometabolic disorders, including the microbially produced histidine metabolite, imidazole propionate (ImP), which has been linked to impaired glucose metabolism [15], and to be associated with the presence and severity of heart failure [16].

Participants in the COCOMO study have been extensively characterized for comorbidities, including research computed tomography (CT)-angiography for evaluating the presence, distribution, and severity of stable CAD [17]. In the present study, we aimed to determine the potential association of gut microbiota alterations with the presence and severity of stable CAD in PWH, and based on these microbiota profiles, we subsequently set out to investigate the association between circulating ImP plasma levels and obstructive CAD.

## METHODS

### Study Population

**Figure 1.**
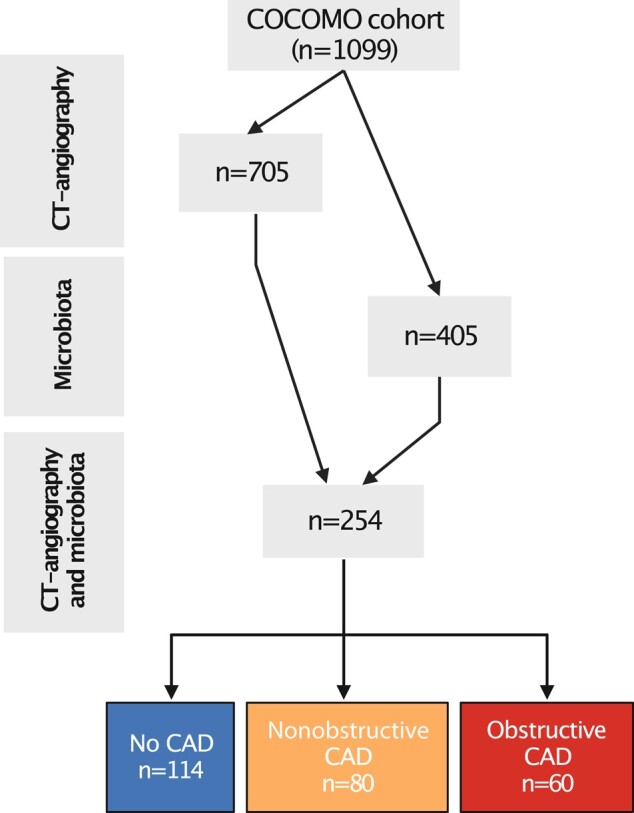
Flow chart of the study design. From the total Copenhagen comorbidity in HIV-infection (COCOMO) cohort, n = 254 participants had availability of both computed tomography (CT) angiography and gut microbiota samples and were included in the present study. Participants were grouped according to the presence of coronary artery disease (CAD) with/out obstructive features. Image was made with BioRender.

The COCOMO study (n = 1099) is a longitudinal study aiming to assess the burden of non-AIDS comorbidities in PWH. Inclusion criteria were a positive HIV test and age older than 18 years. The COCOMO has included more than 40% of the PWH population residing in the Copenhagen area. All COCOMO participants were offered a high-resolution research coronary CT-angiography and were also asked to deliver a stool and plasma sample. Exclusion criteria for coronary CT angiography where reduced kidney function or prior contrast-induced anaphylaxis. From the original cohort, a total of 254 participants were analyzed by both CT-angiography and gut microbiota composition at baseline and included in this cross-sectional substudy (Figure 1) [13, 14, 17]. All participants with available CT-angiography and stool samples were included. Ethical approval was obtained from the Regional Ethics Committee of Copenhagen (H-15017350). Written informed consent was obtained from all participants.

### Multidetector Computed Tomographic Angiography Image Acquisition

As previously described in detail, CT-scans were analyzed in a 17-coronary segment model, with grading of luminal diameter stenosis for each segment of the coronary tree, according to the Society of Cardiovascular Computed Tomography Guidelines using dedicated software (Vitrea 6.7; Vital Images, Inc) [18]. Participants were categorized according to the most severely obstructive coronary artery lesion identified in the coronary tree, into 1 of the following categories: obstructive CAD defined as ≥50% stenosis, nonobstructive CAD defined as 1%–49% stenosis, and no CAD [18].

### Soluble Markers of Inflammation and Plasma Levels of ImP

Plasma samples were collected and stored at −80°C until use. Interleukin 6 (IL-6) was measured by enzyme immunoassays and ImP by liquid chromatography-tandem mass spectrometry, as previously described (https://bevital.no) [14, 19].

### Gut Microbiota Sampling, Sequencing, and Bioinformatics

As previously described in detail, stool samples were stored in collection tubes with DNA stabilized (Stratec Molecular), and DNA was extracted using the PSPSpin Stool DNA-Plus Kit (Stratec Molecular, GmbH), slightly modified by adding a bead-beating step [14]. Libraries were generated from polymerase chain reaction (PCR) amplicons targeting the hypervariable regions V3 and V4 of the 16S rRNA gene. Sequencing was performed at the Norwegian Sequencing Centre (Oslo, Norway), applying the Illumina MiSeq platform and version 3 kit, allowing for 300 bp paired-end reads.

For bioinformatics analyses, paired-end reads were filtered for Illumina Universal Adapters and PhiX, demultiplexed, quality trimmed and merged using bbduk 38.25, je 1.2, cutadapt 1.18, and bbmerge. Denoising to amplicon sequence variants (ASVs), taxonomic classification, and filtering of contaminants and rare ASVs were done with QIIME2 version 2018.8. α diversity and all further analyses were performed on a rarefied (subsampled) dataset with an ASV count of 6247 per sample [14]. Differences in β diversity were assessed by PERMANOVA.

To identify the taxa that were most predictive of ImP plasma levels, we used a random forest model with ImP as dependent variable and bacterial taxa as independent variables using the randomForest package. Internal validity was tested using 200 resamplings (bootstrapping) of the random forest model fitting procedure, and taxa were ranked based on how often they were selected among the top 20 most positive or negative predictive taxa within each resampled dataset. Differentially abundant taxa were calculated using linear discriminant analysis effect size (LEfSe).

### Statistical Analyses

Continuous variables are reported as median and interquartile range and categorical variables as frequency and percentage. Multiple groups were compared with ANOVA followed by *t* tests or Kruskal-Wallis followed by Wilcoxon signed-rank test for continuous data with normal or nonnormal distribution, respectively. χ^2^ or Fisher tests was used for comparison of categorical data. Correlations were tested using Spearman test.

Elevated dysbiosis index was defined as above 75th percentile as previously described for the HIV-related dysbiosis [14]. Associations between CAD-related dysbiosis index, plasma levels of ImP, and obstructive CAD (obstructive CAD versus reference group [nonobstructive CAD and no CAD combined]) were tested using logistic a priori defined regression models adjusted in model 1 for: traditional cardiovascular risk factors (Framingham score), HIV-related factors (duration of HIV, mode of transmission, and nadir of CD4 count), medication potentially influencing microbiota composition or occurrence of CAD (statins, abacavir, and antibiotics), inflammation (serum levels of IL-6) [17]. Model 2 was as model 1 plus adjusted for age and sex. The mediation effect (mean indirect effect) by CAD-related dysbiosis index, plasma levels of ImP and obstructive CAD was investigated using the mediate function included in the R package “Psych”. Statistical significance and confidence intervals (CIs) of the mean indirect effect were computed using bootstrapping method with 1000 iterations. All statistical analyses and data visualizations were done in R (version 3.3.2).

## RESULTS

### Baseline Characteristics

Out of 254 PWH analyzed by both CT-angiography and gut microbiota composition, n = 114 had no CAD, n = 80 had nonobstructive CAD, and n = 60 had obstructive CAD (Figure 1). Demographic and clinical characteristics of participants are presented in Table 1. A comparison of demographic and clinical characteristics of the full COCOMO cohort and our subsample is presented in Supplementary Table 1, showing that participant in the subsample were on average 3.2 years older and had a slightly higher Framingham risk score. The majority was of male sex with a median age of 52 years. The vast majority was virally suppressed on current ART. PWH with obstructive CAD were older, with higher frequency of traditional and HIV-related risk factors compared to PWH with nonobstructive CAD and no CAD.

**Table 1.** Baseline Characteristics of the COCOMO Subsample According to the Presence of Coronary Obstructive Disease

Characteristic	No CAD (n = 114)	Nonobstructive CAD (n = 80)	Obstructive CAD (n = 60)	*P* Value
Age, y	48 (41.8–52.5)	56.1 (48.2–63.0)	60.4 (53.8–68.5)	.004
Sex, male, n (%)	95 (83.3)	74 (92.5)	55 (91.7)	.095
Smoking,^a^ yes, n (%)	67 (58.8)	53 (66.3)	44 73.3)	.229
Hypertension, yes, n (%)	46 (40.3)	41 (51.3)	40 (66.7)	.008
BMI, kg/m^2^	24.1 (21.5–26.9)	24.2 (22.4–27.2)	24.1 (22.8–26)	.519
Framingham risk score	8.4 (4.6–13.9)	15.0 (9.7–22.7)	24.9 (11.6–36.0)	<.001
Metabolic syndrome, yes, n (%)	29 (25.4)	23 (28.7)	31 (51.7)	.010
Use of statins, yes, n (%)	5 (4.6)	8 (10.5)	21 (35.6)	<.001
Mode of transmission, MSM, yes, n (%)	76 (66.6)	62 (77.5)	46 (76.7)	.178
Duration of HIV infection, y	10.9 (5.6–16.6)	14.1 (6.6–21.0)	23.5 (17.9–29.2)	<.001
History of AIDS-defining events, yes, n (%)	15 (13.2)	17 (21.3)	19 (31.7)	.014
CD4 nadir < 200 cells/μL, n (%)	34 (29.8)	40 (50)	33 (55)	.004
Viral load < 50 copies/mL, yes, n (%)	107 (93.8)	79 (98.7)	60 (100)	.044
Current ART treatment, yes, n (%)	113 (99.1)	79 (98.7)	60 (100)	.702
Duration of ART, y	7.2 (3.9–14.3)	12.6 (5.1–18.0)	18.1 (13.8–19.5)	<.001
Use of abacavir, yes, n (%)	31 (27.2)	23 (28.7)	14 (23.3)	.766
IL-6, pg/mL	1.3 (0.9–1.7)	1.7 (1.1–2.5)	2 (1.3–2.6)	<.001
Use of antibiotics, yes, n (%)^b^	20 (17.5)	17 (21.3)	14 (23.3)	.631

Continuous values are shown as median (interquartile range). Characteristics of participants were compared across groups using Kruskal-Wallis test for continuous variables and Fisher test for categorical variables.

Abbreviations: ART, antiretroviral therapy; BMI, body mass index; CAD, coronary artery disease; COCOMO, Copenhagen Comorbidity in HIV Infection Study; HIV, human immunodeficiency virus; IL-6, interleukin 6; MSM, men who have sex with men.

^a^Current or past smoking.

^b^In the 3 months before sampling.

### PWH With Obstructive CAD Have a Specific Microbiota Compositional Shift

**Figure 2.**
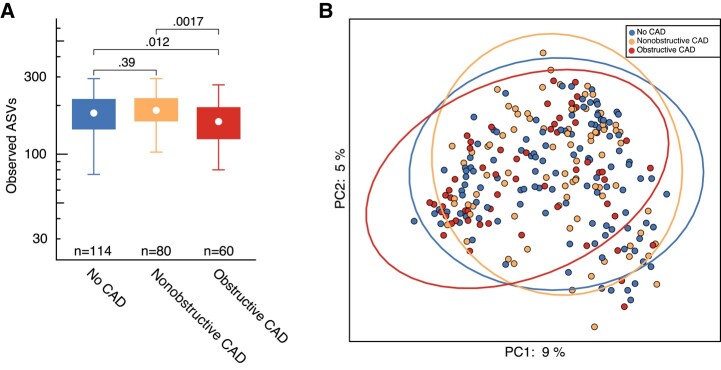
PWH with obstructive CAD have a reduced microbiota richness. α diversity (*A*) measured as number of observed bacterial taxa (ASVs) and β diversity measured as Bray Curtis distances (*B*) PC analysis 1 and PC analysis 2 in PWH without CAD, with nonobstructive CAD, and with obstructive CAD. *P* values were calculated using Wilcoxon rank sum test. Data are represented when appropriated as boxplots: white circle is the median, the lower and upper hinges are the first and third quartiles, the upper whisker extends from the hinge to the largest value no further than 1.5× the interquartile range from the hinge, and the lower whisker extends from the hinge to the smallest value, at most 1. See also Supplementary Figure 1. Abbreviations: ASV, amplicon sequence variants; CAD, coronary artery disease; PC, principal component; PWH, people with human immunodeficiency virus.

The intraindividual α diversity, measured as number of observed bacterial ASVs, was significantly lower in PWH with obstructive CAD compared to both nonobstructive CAD and no CAD (Figure 2*A***)**, and all α diversity measures (Shannon index, observed ASVs, and Faith phylogenetic diversity) showed the same significant trend (Supplementary Figure 1*A–C*). Also, the interindividual β diversity was significantly different in participants with obstructive CAD compared to nonobstructive CAD (*R*^2^ = 0.13, *P* = .004) and no CAD (*R*^2^ = 0.11, *P* = .001). No differences were observed in α or β diversity between PWH with nonobstructive CAD and without CAD (Figure 2*B* and Supplementary Figure 1*D*).

**Figure 3.**
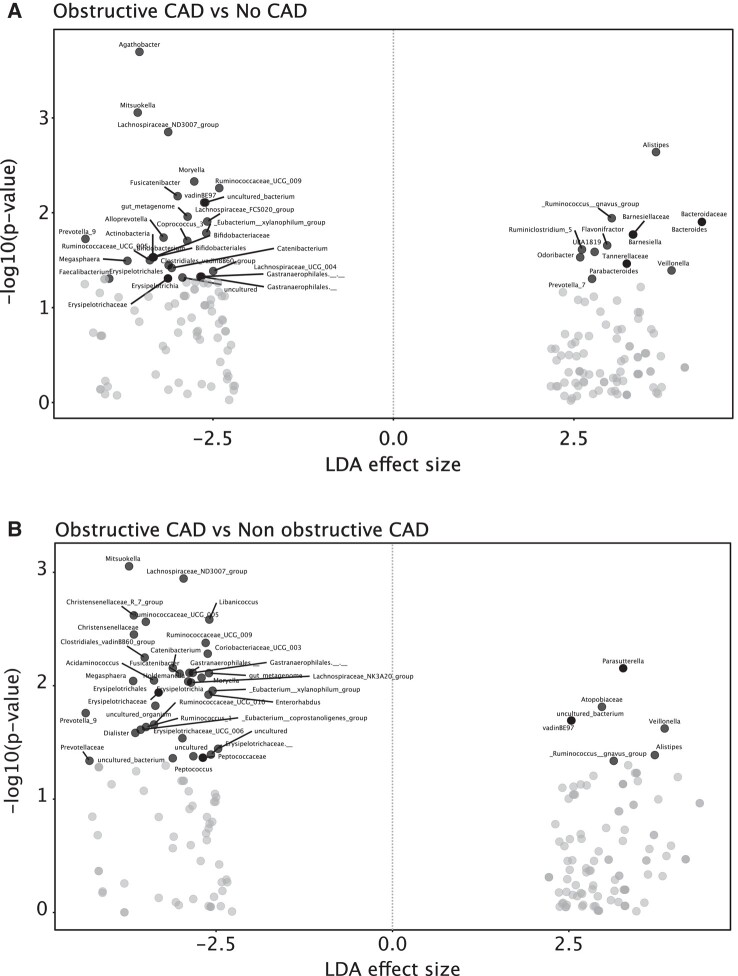
People with human immunodeficiency virus (PWH) with obstructive coronary artery diseases have a shift in microbiota composition. *A* and *B*, Linear discriminant analysis (LDA) effect size of relative taxa abundance in PWH without CAD, with nonobstructive coronary artery disease (no CAD, nonobstructive CAD), and with obstructive CAD. Data are shown as volcano plot. Significant increased or decreased taxa abundances are shown in red or blue and were calculated using linear discriminant analysis of effect size. See also Supplementary Tables 1 and 2 and Supplementary Figure 2.

As shown in Figure 3 and Supplementary Tables 2 and 3, several bacterial genera were differentially abundant in PWH with obstructive CAD compared to nonobstructive CAD and no CAD, including increased relative abundance of *Ruminococcus gnavus* and *Veillonella,* both known producers of ImP, and reduced abundance of several bacterial genera, some with potential for butyrate production. We also observed subtle yet significant differences at the genus level between PWH with nonobstructive CAD and without CAD (Supplementary Figure 2 and Supplementary Table 4).

### A Dysbiosis Index Captures the Gut Microbiota Alterations Associated With Obstructive CAD

Next, we tested the ability of the microbiota to discriminate PWH with obstructive CAD from nonobstructive CAD and participants without CAD. The significantly altered bacteria genera in presence of obstructive CAD were used to define a dysbiosis index (CAD-related dysbiosis index), using a method similar to that for the previously described HIV-index [14]: log_e_([sum of the relative abundances of bacterial taxa upregulated in obstructive CAD]/[sum of the relative abundances of bacterial taxa reduced in obstructive CAD]). This resulted in the following index: Log_e_ ([*Veillonella* + *Ruminococcus gnavus* + *Alistipes*)/(*Prevotella* 9 + *Megashpaera* + *Moryella* + *Catenibacterium* + *Fusicatenibacter* + *Ruminococcacea* UCG 005 + *Ruminococcacea* UCG 009 + *Lachnospiracea* ND3007 group + *Eubacterium xylanophilum* group]). We identified no overlapping genera between CAD-related dysbiosis index and the previously established HIV-related dysbiosis index [14], and the HIV-related dysbiosis index was not related to obstructive CAD or nonobstructive CAD (*P* > .05). The CAD-related dysbiosis index showed a moderate positive correlation with plasma levels of IL-6 (ρ = 0.14, *P* = .03).

### Plasma Levels of ImP Are Elevated in Participants With Obstructive CAD and Associated With Gut Dysbiosis

**Figure 4.**
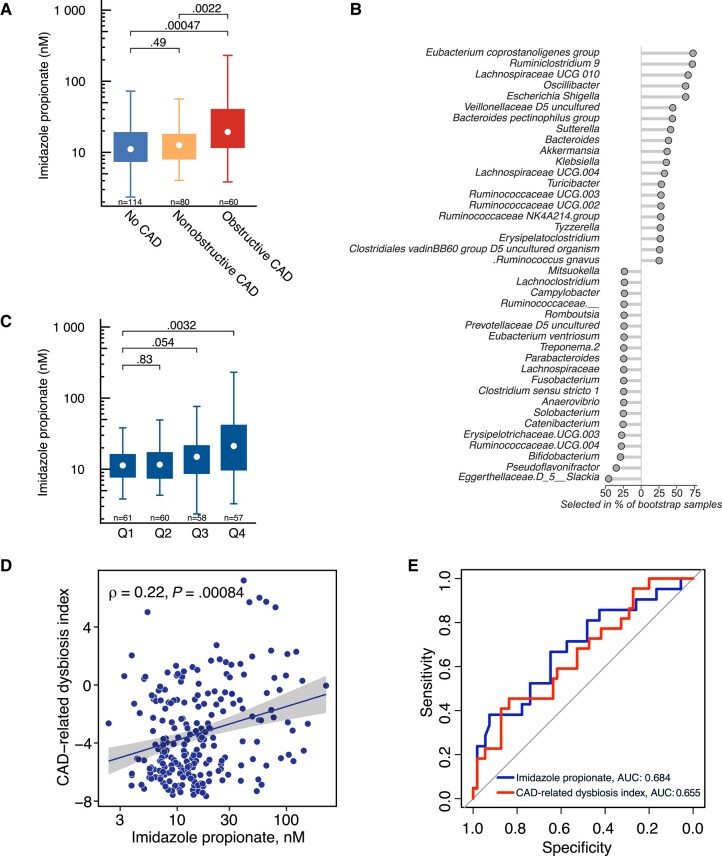
Plasma levels of ImP in relation to dysbiosis and obstructive CAD. *A*, ImP plasma levels in PWH without CAD, with nonobstructive coronary artery disease (no CAD, nonobstructive CAD), and with obstructive CAD. *P* values were calculated using Wilcoxon test. *B*, Random forest plot for the significant genera correlated with ImP. *C*, ImP plasma levels according to CAD dysbiosis index. *P* values were calculated using Wilcoxon test. Data are represented as boxplots: white dot is the median, the lower and upper hinges are the first and third quartiles, the upper whisker extends from the hinge to the largest value no further than 1.5× the interquartile range from the hinge, and the lower whisker extends from the hinge to the smallest value above or equal to 1. *D*, Correlation plot between ImP plasma levels and CAD dysbiosis index. *ρ* and *P* values were calculated using spearman correlation test. *E*, ROC curve for ImP and CAD dysbiosis index in association with obstructive CAD. Abbreviations: AUC, area under the curve; CAD, coronary artery disease; ImP, imidazole propionate; PWH, people with human immunodeficiency virus; ROC, receiver operating characteristic.

Based on the microbial taxa identified in the CAD-related dysbiosis index, with *R. gnavus* and *Veillonella* as 2 of the main microbial producers of ImP [15] and on the newly published data showing ImP to be associated with cardiovascular diseases [16, 20, 21], we next measured plasma levels of ImP in our cohort. ImP plasma levels were significantly higher in PWH with obstructive CAD compared to the other groups; however, they were not higher in those with nonobstructive CAD compared to PWH with no CAD (Figure 4*A*).

Using random forest method with resampling and bootstrap approach, we observed specific bacteria taxa associated with ImP production in obstructive CAD with high internal validity (Figure 4*B*). Among the top 20 associated with ImP we observed taxa that have been previously reported to be associated with ImP plasma levels, including *R. gnavus*, a group of Veillonellacea, and Clostridiales [15].

To evaluate the contribution of an altered microbiota to ImP production, we evaluated ImP plasma levels according to quartiles of the CAD-related dysbiosis index. Increasing ImP plasma levels were associated with a higher degree of dysbiosis, suggesting that CAD-related microbiota alterations could contribute to circulating ImP (Figure 4*C*). Moreover ImP plasma levels correlated with CAD-related dysbiosis index (ρ = 0.22, *P* = .0008; Figure 4*D*).

### Gut Dysbiosis But Not ImP Plasma Levels Are Independently Associated With Obstructive CAD

Finally, we tested the performance of plasma ImP compared to gut dysbiosis in association with obstructive CAD. Receiver operating characteristic (ROC) curves showed approximately similar area under the curve (AUC) for CAD-related dysbiosis index and ImP plasma levels in relation to obstructive CAD (Figure 4*E*). Plasma levels of ImP (> 75th percentile, > 25.1 nM) were associated with higher odds of obstructive CAD in univariable analyses but not in multivariate analysis when adjusting for confounding factors (Table 2). In contrast, after adjusting for traditional cardiovascular risk factors (Framingham score), HIV-related factors (duration of HIV, mode of transmission, and nadir of CD4 count), medication potentially influencing microbiota composition (statins, abacavir, and antibiotics), inflammation (IL-6), age, and sex (model 2), elevated CAD dysbiosis index (> 75th percentile, > −1.06) was significantly associated with obstructive CAD (OR, 2.7; 95% CI, 1.1–7.2; *P* = .048; Table 2). We finally performed a mediation analysis to evaluate the potential mediating effect of ImP on the association between CAD dysbiosis index and the presence of obstructive CAD. CAD dysbiosis index total and direct association was 0.06 (standard error = 0.02, *P* = .002), whereas ImP did not mediate any significant proportion of the association between dysbiosis index and obstructive CAD (*P* = .132).

**Table 2.** Odds Ratio for Presence of Obstructive CAD According to Plasma Levels of ImP or CAD Dysbiosis Index

Model	Factor	OR	(95% CI)	*P* Value
Unadjusted	ImP^a^	2.3	(1.2–4.3)	.010
Adjusted model 1	ImP^a^	1.5	(.6–3.6)	.364
	Framingham risk score	2.9	(1.5–5.3)	<.001
	Use of statins	6.9	(2.5–19.9)	<.001
	Duration of HIV infection, y^b^	5.6	(2.4–13)	<.001
	IL-6, pg/mL	0.9	(.5–1.6)	.865
	Mode of transmission^c^	1.3	(.4–3.4)	.609
	Nadir of CD4^d^	2.3	(.9–5.6)	.072
	Use of abacavir	0.5	(.2–1.2)	.119
	Use of antibiotics	0.5	(.2–1.4)	.180
Adjusted model 2	ImP^a^	1.3	(.5–3.3)	.533
	Framingham risk score	2.3	(1–5)	.041
	Use of statins	6.8	(2.5–18.8)	<.001
	Duration of HIV infection, y^b^	5	(2.1–11.7)	<.001
	IL-6, pg/mL	0.8	(.5–1.5)	.602
	Mode of transmission^c^	1.9	(.5–6.6)	.339
	Nadir of CD4^d^	2.6	(1–6.7)	.044
	Use of abacavir	0.5	(.2–1.2)	.112
	Use of antibiotics	0.5	(.2–1.2)	.112
	Age	16.7	(.6–458.5)	.096
	Male sex	0.5	(.1–2.9)	.420
Unadjusted	CAD-dysbiosis index^a^	4.4	(2.4–8.2)	<.001
Adjusted model 1	CAD-dysbiosis index^a^	3.1	(1.2–7.9)	.020
	Framingham risk score	3.3	(1.6–5.7)	<.001
	Use of statins	6.3	(2.2–17.8)	<.001
	Duration of HIV infection, y^b^	5.1	(2.1–11.7)	<.001
	IL-6, pg/mL	1	(.6–1.6)	.866
	Mode of transmission^c^	2	(.7–5.9)	.233
	Nadir of CD4^d^	2.3	(.9–5.9)	.067
	Use of abacavir	0.5	(.2–1.2)	.142
	Use of antibiotics	0.4	(.2–1.4)	.176
Adjusted model 2	CAD-dysbiosis index^a^	2.7	(1.1–7.2)	.048
	Framingham risk score	2.5	(1.1–5.5)	.027
	Use of statins	6.2	(2.2–17.4)	<.001
	Duration of HIV infection, y^b^	4.6	(2–10.8)	<.001
	IL-6, pg/mL	0.8	(.5–1.5)	.639
	Mode of transmission^c^	2.6	(.6–10.7)	.173
	Nadir of CD4^d^	2.6	(1–6.7)	.046
	Use of abacavir	0.5	(.2–1.3)	.131
	Use of antibiotics	0.5	(.2–1.5)	.235
	Age	10.5	(.4–290)	.162
	Male sex	0.5	(.1–3.4)	.503

Abbreviations: ImP, imidazole propionate; CAD, coronary artery disease; CI, confidence interval; HIV, human immunodeficiency virus; IL-6, interleukin 6; OR, odds ratio.

^a^Highest quartile versus the others.

^b^Every 5 years.

^c^Men having sex with men versus other.

^d^Less than 200 cells/μL.

## DISCUSSION

The aim of this study was to determine the potential impact of gut microbiota alterations on the presence and severity of CAD in PWH. Our findings can be summarized as follows: (1) participants with obstructive CAD had an altered gut microbiota with lower α diversity and higher β diversity compared no nonobstructive CAD and no CAD; (2) participants with obstructive CAD also had distinctive compositional microbiota shift, including increased relative abundance of *R. gnavus* and *Veillonella,* taxa that have been strongly associated with ImP; (3) plasma levels of ImP were elevated in participants with obstructive CAD and correlated significantly with gut microbiota alterations; and (4) gut microbiota alterations rather than ImP plasma levels were independently associated with obstructive CAD after adjustment for traditional and HIV-related risk factors.

Although gut microbiota alterations in PWH have been extensively studied in the last decade [5–10], few studies have attempted to link gut dysbiosis to comorbidities, including CAD. A small substudy from the HIV-HEART trial compared 30 PWH with CAD and 30 PWH without CAD, finding a lower α diversity in PWH with CAD, whereas compositional changes were mostly related to MSM status [22], in line with several microbiota studies in PWH [12].

In the present cohort, we recently established an HIV-related microbiota index adjusted for MSM status by comparison with a matched PREP cohort [14]. As this HIV-related microbiota index was closely associated with the metabolic syndrome, a separate question of the present work was to investigate whether this would translate into increased risk of CAD. However, the HIV-related microbiota index was not related to obstructive CAD, and except for low α diversity, and possibly reduced capacity for butyrate production, we identified no overlapping features between the previously reported HIV-related and the present CAD-related microbiota profile.

A likely interpretation is that microbiota traits associated with obstructive CAD in PWH are not HIV specific. In the general population, alterations in microbiota composition with reduced potential for butyrate production, disruption of the gut barrier, microbial translocation, and subsequent inflammation have all been associated with the development of cardiometabolic disorders, as previously reviewed [4]. However, there are few reports focusing specifically on microbiota alterations related to symptomatic or obstructive CAD in the general population.

Interestingly, one such study reported increased abundance of 3 bacterial genera including *R. gnavus* in patients with advanced CAD [23], in line with the first metagenomics study in the field reporting increased abundance of *R. gnavus* in patients with atherosclerotic cardiovascular disease [24]. Another bacterial taxa that was enriched in obstructive CAD was *Veillonella*, which has not been frequently reported in studies related to CAD, although it was detected in the majority of atherosclerotic plaque samples as well as the oral cavity in a study focusing on gut, oral, and plaque microbiota [25].

Because *R. gnavus* and *Veillonella* have been reported to be strongly associated with ImP, we hypothesized that circulating ImP plasma levels could be a potential biomarker of obstructive CAD, reflecting the underlying gut dysbiosis. Indeed, ImP plasma levels were higher in PWH with obstructive CAD compared to both participants with nonobstructive CAD and no CAD. Our findings are in line with a recent report of ImP being associated with carotid atherosclerosis in women with HIV [20], and extend these data by linking circulating ImP plasma levels both to disease-related dysbiosis as well as obstructive CAD in PWH irrespective of sex or mode of transmission.

However, although *R. gnavus* and *Veillonella* correlated positively with circulating ImP, several microbes not involved in the dysbiosis index correlated even stronger with ImP plasma levels. Furthermore, when correcting for traditional and HIV-related risk factors, as well as systemic inflammation, gut dysbiosis but not ImP plasma levels remained independently associated with obstructive CAD. Moreover, ImP did not mediate a significant proportion of the association between gut dysbiosis and obstructive CAD, suggesting that most of the effects of the gut microbiota go beyond ImP. Of potential relevance, the dysbiosis index correlated positively with systemic IL-6 levels, although the association was moderate. Animal models have demonstrated a causal role of a dysbiotic microbiota in obstructive CAD, although the underlying mechanisms are not well understood [26]. Hence, ImP could act as a potential biomarker of specific taxa such as *R. gnavus* and *Veillonella,* which could be involved in the atherosclerotic process through separate mechanisms. Of note, *R. gnavus* has been linked to several inflammatory diseases [27–29], possibly through its ability to produce proinflammatory polysaccharides [30], whereas *Veillonella* has been linked to various fibrotic conditions, as also reported by our group in primary sclerosing cholangitis [31] and pulmonary sequela after coronavirus disease 2019 (COVID-19) [32].

The present study has some limitations. First, due to cross-sectional design, conclusions about causality cannot be drawn. Second, differences in age and sex may explain part of the findings, although possible confounding was reduced by adjustment in multivariable regression. Third, due to the low number of viremic participants, our results may not be translated to settings with higher prevalence of uncontrolled viral replication. Our study has obvious strengths, being the largest cohort of PWH with microbiota samples in a carefully characterized population, well controlled for confounding factors.

In conclusion, PWH with obstructive CAD had distinct gut microbiota profiles and elevated ImP plasma levels compared to PWH with nonobstructive CAD and no CAD. Plasma ImP seems to capture aspects of the underlying microbiota alterations related to obstructive CAD, and is therefore a promising circulating biomarker reflecting gut dysbiosis, although it is not able to replace dysbiosis in predicting obstructive CAD. Future studies from this longitudinal cohort will determine whether the CAD-related dysbiosis and related metabolites, including ImP, are predictive of future cardiovascular events.

## Supplementary Data

Supplementary materials are available at *The Journal of Infectious Diseases* online (http://jid.oxfordjournals.org/). Supplementary materials consist of data provided by the author that are published to benefit the reader. The posted materials are not copyedited. The contents of all supplementary data are the sole responsibility of the authors. Questions or messages regarding errors should be addressed to the author.

## Supplementary Material

jiad604_Supplementary_Data

## Notes

***Acknowledgments.*** We thank all the study participants for their participation. We thank the staff at the Department of Infectious Diseases at Rigshospitalet and at Hvidovre Hospital for their dedicated participation.

***Disclaimer.*** The study was designed, conducted, analyzed, and written by the authors without involvement of any commercial party.

***Data availability.*** Data related to this study can be found in the main text and Supplementary Material but raw data are subject to restrictions based on approvals from the ethical committee and the Danish data protection agency and may not be shared publicly. The data may be accessed at our institution on reasonable request to the corresponding author.

***Financial support*.** This work was supported by Rigshospitalet Research Council, Region Hovedstaden; the Lundbeck Foundation; the Novo Nordisk Foundation; the South-Eastern Norway Regional Health Authority (grant number 2016004); and the Research Council of Norway (grant number 240787/F20 and 287242).
