# Supplementary material for: Gut Microbiota Alterations and Circulating Imidazole Propionate Levels Are Associated With Obstructive Coronary Artery Disease in People With HIV
Source: J Infect Dis. 2024 Jan 9;229(3):898–907. doi: 10.1093/infdis/jiad604 (PMC10938217; doi:10.1093/infdis/jiad604)
Supplement: jiad604_Supplementary_Data [file jiad604_supplementary_data.zip › Supplementary Figure Legends.docx]

**Supplementary Figure 1. PLWH with obstructive coronary artery diseases have a reduce alpha diversity.**Alpha diversity measured as (**A**) Shannon entropy index, (**B**) Observed Amplicon sequence variants (ASVs) and (**C**) Faith phylogenetic diversity in PLWH without (no CAD), with non-obstructive coronary artery disease (Non−obstructive CAD) and with obstructive CAD. **D.**Bray Curtis distances, PCoA1 and PCoA3 in PLWH without, with non-obstructive coronary artery disease (no CAD, Non−obstructive CAD) and with obstructive CAD. P-value were calculated using Wilcoxon rank sum test. Data are represented as boxplots: white dot is the median, the lower and upper hinges are the first and third quartiles, the upper whisker extends from the hinge to the largest value no further than 1.5× the interquartile range (IQR) from the hinge, and the lower whisker extends from the hinge to the smallest value above or equal to 1.

**Supplementary Figure 2. PLWH with coronary artery diseases have a shift in microbiota composition.** Linear discriminant analysis (LDA) effect size of relative taxa abundance in PLWH without and with non-obstructive coronary artery disease (no CAD, Non−obstructive CAD). Data are shown as Volcano plot. Significantly increased or decreased taxa abundances shown in red or blue were calculated using linear discriminant analysis of effect size. See also Supplementary table 3.
