# Supplementary material for: Gut Microbiota Alterations and Circulating Imidazole Propionate Levels Are Associated With Obstructive Coronary Artery Disease in People With HIV
Source: J Infect Dis. 2024 Jan 9;229(3):898–907. doi: 10.1093/infdis/jiad604 (PMC10938217; doi:10.1093/infdis/jiad604)
Supplement: jiad604_Supplementary_Data [file jiad604_supplementary_data.zip › Supplementary tables HIV IMPr2.docx]

| *Calculated for entire population irrespective of age |  | **Study population**  **N=254** | **COCOMO participants who did not participate**  **N=845** | **P-value** |
| --- | --- | --- | --- | --- |
| Age, years |  | 52.5 (46.4-61.0) | 49.3 (41.9-56.7) | <0.001 |
| Sex, male, n (%) |  | 224 (88.2) | 713 (84.4) | 0.161 |
| Smoking*, yes, n (%) |  | 164 (64.6) | 561 (66.4) | >0.99 |
| Hypertension, yes, n (%) |  | 127 (50.0) | 324 (39.5) | 0.014 |
| BMI kg/m^2^ |  | 24.1 (22.0-26.5) | 24.7 (22.5-27.3) | 0.041 |
| Framingham risk score |  | 12.4 (6.7-23.2) | 10 (4.4-19.1) | 0.002 |
| Metabolic syndrome, yes, n (%) |  | 83 (32.7) | 238 (28.2) | 0.260 |
| Use of statins, yes, n (%) |  | 34 (13.4) | 108 (12.8) | >0.99 |
| Mode of transmission: MSM, yes, n (%) |  | 184 (72.4) | 591 (69.9) | 0.591 |
| Duration of HIV infection, years |  | 14.2 (6.9-22.0) | 13.2 (6.7-20.7) | 0.224 |
| History of AIDS defining events, yes, n (%) |  | 51 (20.1) | 144 (17.0) | 0.554 |
| CD4 nadir < 200 cells, n (%) |  | 107 (42.1) | 336 (39.8) | 0.851 |
| Viral load < 50 copies/ml, yes, n (%) |  | 246 (96.9) | 784 (92.8) | 0.108 |
| Current ART treatment, yes, n (%) |  | 252 (99.2) | 827 (97.9) | 0.408 |
| Duration of ART, years |  | 12.9 (5.1-18.0) | 10 (5.3-16.8) | 0.164 |
| IL-6, pg/mL |  | 3.4 (2.5-4.7) | 3.2 (2.3-4.7) | 0.527 |

**Supplementary table 1. Baseline characteristics of the full COCOMO cohort according to the presence of coronary obstructive disease (CAD).** Continuous values are shown as median (IQR). Characteristics of participants were compared across groups using Kruskal–Wallis test for continuous variables and Fisher test for categorical variables. BMI: body mass index; ART: antiretroviral therapy; MSM: men who have sex with men. *Current or past smoking.** in the 3 months before sampling.

**Supplementary table 2.** Linear discriminant analysis (LDA) effect size of relative significant taxa abundance in PLWH with obstructive CAD vs No CAD. Significant taxa abundance was calculated using linear discriminant analysis of effect size.

| **Taxa** | **LDA** | **P-value** |
| --- | --- | --- |
| D_0__Bacteria.D_1__Bacteroidetes.D_2__Bacteroidia.D_3__Bacteroidales.D_4__Prevotellaceae.D_5__Prevotella_9 | -4.265814307 | 0.01883015 |
| D_0__Bacteria.D_1__Firmicutes.D_2__Clostridia.D_3__Clostridiales.D_4__Ruminococcaceae.D_5__Faecalibacterium | -3.936509631 | 0.04941763 |
| D_0__Bacteria.D_1__Firmicutes.D_2__Negativicutes.D_3__Selenomonadales.D_4__Veillonellaceae.D_5__Megasphaera | -3.684850887 | 0.0320848 |
| D_0__Bacteria.D_1__Firmicutes.D_2__Negativicutes.D_3__Selenomonadales.D_4__Veillonellaceae.D_5__Mitsuokella | -3.542032974 | 0.00087887 |
| D_0__Bacteria.D_1__Firmicutes.D_2__Clostridia.D_3__Clostridiales.D_4__Lachnospiraceae.D_5__Agathobacter | -3.517935914 | 0.00020139 |
| D_0__Bacteria.D_1__Firmicutes.D_2__Clostridia.D_3__Clostridiales.D_4__Ruminococcaceae.D_5__Ruminococcaceae_UCG_005 | -3.373957613 | 0.0316195 |
| D_0__Bacteria.D_1__Actinobacteria.D_2__Actinobacteria | -3.333432553 | 0.0294287 |
| D_0__Bacteria.D_1__Actinobacteria.D_2__Actinobacteria.D_3__Bifidobacteriales.D_4__Bifidobacteriaceae.D_5__Bifidobacterium | -3.328916269 | 0.0294287 |
| D_0__Bacteria.D_1__Actinobacteria.D_2__Actinobacteria.D_3__Bifidobacteriales.D_4__Bifidobacteriaceae | -3.327572017 | 0.0294287 |
| D_0__Bacteria.D_1__Actinobacteria.D_2__Actinobacteria.D_3__Bifidobacteriales | -3.320698799 | 0.0294287 |
| D_0__Bacteria.D_1__Bacteroidetes.D_2__Bacteroidia.D_3__Bacteroidales.D_4__Prevotellaceae.D_5__Alloprevotella | -3.183942657 | 0.01825879 |
| D_0__Bacteria.D_1__Firmicutes.D_2__Erysipelotrichia | -3.123126191 | 0.04918358 |
| D_0__Bacteria.D_1__Firmicutes.D_2__Erysipelotrichia.D_3__Erysipelotrichales.D_4__Erysipelotrichaceae | -3.123126191 | 0.04918358 |
| D_0__Bacteria.D_1__Firmicutes.D_2__Erysipelotrichia.D_3__Erysipelotrichales | -3.123126191 | 0.04918358 |
| D_0__Bacteria.D_1__Firmicutes.D_2__Clostridia.D_3__Clostridiales.D_4__Lachnospiraceae.D_5__Lachnospiraceae_ND3007_group | -3.119668976 | 0.00140985 |
| D_0__Bacteria.D_1__Firmicutes.D_2__Clostridia.D_3__Clostridiales.D_4__Clostridiales_vadinBB60_group | -3.11396873 | 0.0356857 |
| D_0__Bacteria.D_1__Firmicutes.D_2__Erysipelotrichia.D_3__Erysipelotrichales.D_4__Erysipelotrichaceae.D_5__Catenibacterium | -3.071375962 | 0.03813711 |
| D_0__Bacteria.D_1__Firmicutes.D_2__Clostridia.D_3__Clostridiales.D_4__Lachnospiraceae.D_5__Fusicatenibacter | -2.988847333 | 0.00668238 |
| D_0__Bacteria.D_1__Bacteroidetes.D_2__Bacteroidia.D_3__Bacteroidales.D_4__Prevotellaceae.D_5__uncultured | -2.922637587 | 0.04806864 |
| D_0__Bacteria.D_1__Firmicutes.D_2__Clostridia.D_3__Clostridiales.D_4__Lachnospiraceae.D_5__Coprococcus_3 | -2.855169905 | 0.01974702 |
| D_0__Bacteria.D_1__Firmicutes.D_2__Clostridia.D_3__Clostridiales.D_4__Clostridiales_vadinBB60_group.D_5__gut_metagenome | -2.850794968 | 0.01098464 |
| D_0__Bacteria.D_1__Firmicutes.D_2__Clostridia.D_3__Clostridiales.D_4__Lachnospiraceae.D_5__Moryella | -2.75899401 | 0.0046756 |
| D_0__Bacteria.D_1__Cyanobacteria.D_2__Melainabacteria.D_3__Gastranaerophilales.__.__ | -2.674080127 | 0.0471165 |
| D_0__Bacteria.D_1__Cyanobacteria.D_2__Melainabacteria.D_3__Gastranaerophilales.__ | -2.659076643 | 0.0471165 |
| D_0__Bacteria.D_1__Lentisphaerae.D_2__Lentisphaeria.D_3__Victivallales.D_4__vadinBE97 | -2.621812202 | 0.00779987 |
| D_0__Bacteria.D_1__Lentisphaerae.D_2__Lentisphaeria.D_3__Victivallales.D_4__vadinBE97.D_5__uncultured_bacterium | -2.605185959 | 0.00779987 |
| D_0__Bacteria.D_1__Firmicutes.D_2__Clostridia.D_3__Clostridiales.D_4__Lachnospiraceae.D_5___Eubacterium__xylanophilum_group | -2.592264293 | 0.01635489 |
| D_0__Bacteria.D_1__Firmicutes.D_2__Clostridia.D_3__Clostridiales.D_4__Lachnospiraceae.D_5__Lachnospiraceae_FCS020_group | -2.579254586 | 0.01236458 |
| D_0__Bacteria.D_1__Firmicutes.D_2__Clostridia.D_3__Clostridiales.D_4__Lachnospiraceae.D_5__Lachnospiraceae_UCG_004 | -2.49621066 | 0.04117176 |
| D_0__Bacteria.D_1__Firmicutes.D_2__Clostridia.D_3__Clostridiales.D_4__Ruminococcaceae.D_5__Ruminococcaceae_UCG_009 | -2.412113206 | 0.00549161 |
| D_0__Bacteria.D_1__Bacteroidetes.D_2__Bacteroidia.D_3__Bacteroidales.D_4__Marinifilaceae.D_5__Odoribacter | 2.586776501 | 0.02945878 |
| D_0__Bacteria.D_1__Firmicutes.D_2__Clostridia.D_3__Clostridiales.D_4__Ruminococcaceae.D_5__Ruminiclostridium_5 | 2.6113225 | 0.02436235 |
| D_0__Bacteria.D_1__Bacteroidetes.D_2__Bacteroidia.D_3__Bacteroidales.D_4__Prevotellaceae.D_5__Prevotella_7 | 2.751136569 | 0.04965826 |
| D_0__Bacteria.D_1__Firmicutes.D_2__Clostridia.D_3__Clostridiales.D_4__Ruminococcaceae.D_5__UBA1819 | 2.78687478 | 0.02582299 |
| D_0__Bacteria.D_1__Firmicutes.D_2__Clostridia.D_3__Clostridiales.D_4__Ruminococcaceae.D_5__Flavonifractor | 2.960437939 | 0.02206065 |
| D_0__Bacteria.D_1__Firmicutes.D_2__Clostridia.D_3__Clostridiales.D_4__Lachnospiraceae.D_5___Ruminococcus__gnavus_group | 3.024098132 | 0.01140634 |
| D_0__Bacteria.D_1__Bacteroidetes.D_2__Bacteroidia.D_3__Bacteroidales.D_4__Tannerellaceae | 3.231826114 | 0.03436848 |
| D_0__Bacteria.D_1__Bacteroidetes.D_2__Bacteroidia.D_3__Bacteroidales.D_4__Tannerellaceae.D_5__Parabacteroides | 3.231826114 | 0.03436848 |
| D_0__Bacteria.D_1__Bacteroidetes.D_2__Bacteroidia.D_3__Bacteroidales.D_4__Barnesiellaceae | 3.316602397 | 0.01695461 |
| D_0__Bacteria.D_1__Bacteroidetes.D_2__Bacteroidia.D_3__Bacteroidales.D_4__Barnesiellaceae.D_5__Barnesiella | 3.321896402 | 0.01695461 |
| D_0__Bacteria.D_1__Bacteroidetes.D_2__Bacteroidia.D_3__Bacteroidales.D_4__Rikenellaceae.D_5__Alistipes | 3.636572758 | 0.00229153 |
| D_0__Bacteria.D_1__Firmicutes.D_2__Negativicutes.D_3__Selenomonadales.D_4__Veillonellaceae.D_5__Veillonella | 3.852014188 | 0.04053526 |
| D_0__Bacteria.D_1__Bacteroidetes.D_2__Bacteroidia.D_3__Bacteroidales.D_4__Bacteroidaceae | 4.27246425 | 0.01248337 |
| D_0__Bacteria.D_1__Bacteroidetes.D_2__Bacteroidia.D_3__Bacteroidales.D_4__Bacteroidaceae.D_5__Bacteroides | 4.27246425 | 0.01248337 |

**Supplementary table 3.** Linear discriminant analysis (LDA) effect size of relative significant taxa abundance in PLWH with obstructive CAD vs CAD. Significant taxa abundance was calculated using linear discriminant analysis of effect size.

| **Taxa** | **LDA** | **P-value** |
| --- | --- | --- |
| D_0__Bacteria.D_1__Bacteroidetes.D_2__Bacteroidia.D_3__Bacteroidales.D_4__Prevotellaceae.D_5__Prevotella_9 | -4.3477796 | 0.01743153 |
| D_0__Bacteria.D_1__Bacteroidetes.D_2__Bacteroidia.D_3__Bacteroidales.D_4__Prevotellaceae | -4.2941827 | 0.04592179 |
| D_0__Bacteria.D_1__Firmicutes.D_2__Negativicutes.D_3__Selenomonadales.D_4__Veillonellaceae.D_5__Mitsuokella | -3.7331092 | 0.00088529 |
| D_0__Bacteria.D_1__Firmicutes.D_2__Negativicutes.D_3__Selenomonadales.D_4__Veillonellaceae.D_5__Megasphaera | -3.6752299 | 0.00908208 |
| D_0__Bacteria.D_1__Firmicutes.D_2__Clostridia.D_3__Clostridiales.D_4__Christensenellaceae.D_5__Christensenellaceae_R_7_group | -3.6658988 | 0.00239608 |
| D_0__Bacteria.D_1__Firmicutes.D_2__Clostridia.D_3__Clostridiales.D_4__Christensenellaceae | -3.6646044 | 0.00353486 |
| D_0__Bacteria.D_1__Firmicutes.D_2__Negativicutes.D_3__Selenomonadales.D_4__Veillonellaceae.D_5__Dialister | -3.6444643 | 0.02606849 |
| D_0__Bacteria.D_1__Firmicutes.D_2__Clostridia.D_3__Clostridiales.D_4__Ruminococcaceae.D_5___Eubacterium__coprostanoligenes_group | -3.5686169 | 0.02443503 |
| D_0__Bacteria.D_1__Firmicutes.D_2__Clostridia.D_3__Clostridiales.D_4__Clostridiales_vadinBB60_group | -3.5113154 | 0.00564453 |
| D_0__Bacteria.D_1__Firmicutes.D_2__Clostridia.D_3__Clostridiales.D_4__Ruminococcaceae.D_5__Ruminococcaceae_UCG_005 | -3.4958793 | 0.00273076 |
| D_0__Bacteria.D_1__Firmicutes.D_2__Clostridia.D_3__Clostridiales.D_4__Ruminococcaceae.D_5__Ruminococcus_1 | -3.4920717 | 0.02312988 |
| D_0__Bacteria.D_1__Firmicutes.D_2__Clostridia.D_3__Clostridiales.D_4__Ruminococcaceae.D_5__Ruminococcaceae_UCG_010 | -3.3798575 | 0.02198188 |
| D_0__Bacteria.D_1__Firmicutes.D_2__Negativicutes.D_3__Selenomonadales.D_4__Acidaminococcaceae.D_5__Acidaminococcus | -3.3794667 | 0.00899164 |
| D_0__Bacteria.D_1__Firmicutes.D_2__Clostridia.D_3__Clostridiales.D_4__Clostridiales_vadinBB60_group.D_5__uncultured_organism | -3.3603664 | 0.01502702 |
| D_0__Bacteria.D_1__Firmicutes.D_2__Erysipelotrichia | -3.3146678 | 0.0114808 |
| D_0__Bacteria.D_1__Firmicutes.D_2__Erysipelotrichia.D_3__Erysipelotrichales.D_4__Erysipelotrichaceae | -3.3146678 | 0.0114808 |
| D_0__Bacteria.D_1__Firmicutes.D_2__Erysipelotrichia.D_3__Erysipelotrichales | -3.3146678 | 0.0114808 |
| D_0__Bacteria.D_1__Firmicutes.D_2__Clostridia.D_3__Clostridiales.D_4__Clostridiales_vadinBB60_group.D_5__uncultured_bacterium | -3.1137618 | 0.0435927 |
| D_0__Bacteria.D_1__Firmicutes.D_2__Erysipelotrichia.D_3__Erysipelotrichales.D_4__Erysipelotrichaceae.D_5__Catenibacterium | -3.1133478 | 0.00698168 |
| D_0__Bacteria.D_1__Firmicutes.D_2__Clostridia.D_3__Clostridiales.D_4__Lachnospiraceae.D_5__Fusicatenibacter | -3.0166489 | 0.00782886 |
| D_0__Bacteria.D_1__Firmicutes.D_2__Erysipelotrichia.D_3__Erysipelotrichales.D_4__Erysipelotrichaceae.D_5__Erysipelotrichaceae_UCG_006 | -2.9770609 | 0.02904908 |
| D_0__Bacteria.D_1__Firmicutes.D_2__Clostridia.D_3__Clostridiales.D_4__Lachnospiraceae.D_5__Lachnospiraceae_ND3007_group | -2.9605205 | 0.00113486 |
| D_0__Bacteria.D_1__Firmicutes.D_2__Erysipelotrichia.D_3__Erysipelotrichales.D_4__Erysipelotrichaceae.D_5__Holdemanella | -2.8956383 | 0.00917988 |
| D_0__Bacteria.D_1__Cyanobacteria.D_2__Melainabacteria.D_3__Gastranaerophilales.__ | -2.8781697 | 0.0076975 |
| D_0__Bacteria.D_1__Firmicutes.D_2__Clostridia.D_3__Clostridiales.D_4__Lachnospiraceae.D_5__Lachnospiraceae_NK3A20_group | -2.8539572 | 0.00938421 |
| D_0__Bacteria.D_1__Cyanobacteria.D_2__Melainabacteria.D_3__Gastranaerophilales.__.__ | -2.8306391 | 0.0076975 |
| D_0__Bacteria.D_1__Firmicutes.D_2__Clostridia.D_3__Clostridiales.D_4__Ruminococcaceae.D_5__uncultured | -2.822008 | 0.04188586 |
| D_0__Bacteria.D_1__Firmicutes.D_2__Clostridia.D_3__Clostridiales.D_4__Lachnospiraceae.D_5__Moryella | -2.7057578 | 0.00849032 |
| D_0__Bacteria.D_1__Firmicutes.D_2__Clostridia.D_3__Clostridiales.D_4__Peptococcaceae.D_5__Peptococcus | -2.6888205 | 0.04314972 |
| D_0__Bacteria.D_1__Firmicutes.D_2__Clostridia.D_3__Clostridiales.D_4__Peptococcaceae | -2.6856616 | 0.04314972 |
| D_0__Bacteria.D_1__Firmicutes.D_2__Clostridia.D_3__Clostridiales.D_4__Ruminococcaceae.D_5__Ruminococcaceae_UCG_009 | -2.6478121 | 0.00419171 |
| D_0__Bacteria.D_1__Actinobacteria.D_2__Coriobacteriia.D_3__Coriobacteriales.D_4__Atopobiaceae.D_5__Coriobacteriaceae_UCG_003 | -2.618165 | 0.0052224 |
| D_0__Bacteria.D_1__Actinobacteria.D_2__Coriobacteriia.D_3__Coriobacteriales.D_4__Eggerthellaceae.D_5__Enterorhabdus | -2.6079069 | 0.01199482 |
| D_0__Bacteria.D_1__Firmicutes.D_2__Clostridia.D_3__Clostridiales.D_4__Clostridiales_vadinBB60_group.D_5__gut_metagenome | -2.6002271 | 0.00774664 |
| D_0__Bacteria.D_1__Actinobacteria.D_2__Coriobacteriia.D_3__Coriobacteriales.D_4__Atopobiaceae.D_5__Libanicoccus | -2.59562 | 0.00261051 |
| D_0__Bacteria.D_1__Firmicutes.D_2__Erysipelotrichia.D_3__Erysipelotrichales.D_4__Erysipelotrichaceae.__ | -2.5733857 | 0.04050376 |
| D_0__Bacteria.D_1__Firmicutes.D_2__Clostridia.D_3__Clostridiales.D_4__Lachnospiraceae.D_5___Eubacterium__xylanophilum_group | -2.5492304 | 0.01107339 |
| D_0__Bacteria.D_1__Actinobacteria.D_2__Coriobacteriia.D_3__Coriobacteriales.D_4__Eggerthellaceae.D_5__uncultured | -2.4712858 | 0.03599913 |
| D_0__Bacteria.D_1__Lentisphaerae.D_2__Lentisphaeria.D_3__Victivallales.D_4__vadinBE97 | 2.5320025 | 0.02027044 |
| D_0__Bacteria.D_1__Lentisphaerae.D_2__Lentisphaeria.D_3__Victivallales.D_4__vadinBE97.D_5__uncultured_bacterium | 2.53680624 | 0.02027044 |
| D_0__Bacteria.D_1__Actinobacteria.D_2__Coriobacteriia.D_3__Coriobacteriales.D_4__Atopobiaceae | 2.96828247 | 0.01535659 |
| D_0__Bacteria.D_1__Proteobacteria.D_2__Gammaproteobacteria.D_3__Betaproteobacteriales.D_4__Burkholderiaceae.D_5__Parasutterella | 3.26938673 | 0.0070165 |
| D_0__Bacteria.D_1__Bacteroidetes.D_2__Bacteroidia.D_3__Bacteroidales.D_4__Rikenellaceae.D_5__Alistipes | 3.71769849 | 0.04089858 |
| D_0__Bacteria.D_1__Firmicutes.D_2__Negativicutes.D_3__Selenomonadales.D_4__Veillonellaceae.D_5__Veillonella | 3.85725046 | 0.02383658 |

**Supplementary table 4.** Linear discriminant analysis (LDA) effect size of relative significant taxa abundance in PLWH with CAD vs no CAD. Significant taxa abundance was calculated using linear discriminant analysis of effect size.

| **Taxa** | **LDA** | **P-value** |
| --- | --- | --- |
| D_0__Bacteria.D_1__Firmicutes.D_2__Clostridia.D_3__Clostridiales.D_4__Lachnospiraceae | -4.1663537 | 0.02136379 |
| D_0__Bacteria.D_1__Firmicutes.D_2__Negativicutes.D_3__Selenomonadales.D_4__Acidaminococcaceae.D_5__Phascolarctobacterium | -3.8296037 | 0.04541809 |
| D_0__Bacteria.D_1__Firmicutes.D_2__Clostridia.D_3__Clostridiales.D_4__Lachnospiraceae.D_5__Agathobacter | -3.563805 | 0.00185309 |
| D_0__Bacteria.D_1__Firmicutes.D_2__Bacilli | -2.765683 | 0.03134919 |
| D_0__Bacteria.D_1__Firmicutes.D_2__Bacilli.D_3__Lactobacillales | -2.765683 | 0.03134919 |
| D_0__Bacteria.D_1__Firmicutes.D_2__Bacilli.D_3__Lactobacillales.D_4__Streptococcaceae | -2.765683 | 0.03134919 |
| D_0__Bacteria.D_1__Firmicutes.D_2__Bacilli.D_3__Lactobacillales.D_4__Streptococcaceae.D_5__Streptococcus | -2.765683 | 0.03134919 |
| D_0__Bacteria.D_1__Firmicutes.D_2__Clostridia.D_3__Clostridiales.D_4__Lachnospiraceae.D_5__Lachnospiraceae_UCG_010 | -2.4789615 | 0.01463521 |
| D_0__Bacteria.D_1__Firmicutes.D_2__Clostridia.D_3__Clostridiales.D_4__Ruminococcaceae.D_5__Ruminococcus_2 | 3.57859266 | 0.02977336 |
| D_0__Bacteria.D_1__Firmicutes.D_2__Clostridia.D_3__Clostridiales.D_4__Christensenellaceae.D_5__Christensenellaceae_R_7_group | 3.74282177 | 0.02732954 |
| D_0__Bacteria.D_1__Firmicutes.D_2__Clostridia.D_3__Clostridiales.D_4__Christensenellaceae | 3.7436721 | 0.02592193 |
