# Supplementary figures and images for: Gut Microbiota Alterations and Circulating Imidazole Propionate Levels Are Associated With Obstructive Coronary Artery Disease in People With HIV
Source: J Infect Dis. 2024 Jan 9;229(3):898–907. doi: 10.1093/infdis/jiad604 (PMC10938217; doi:10.1093/infdis/jiad604)

### Non-obstructive CAD vs No CAD

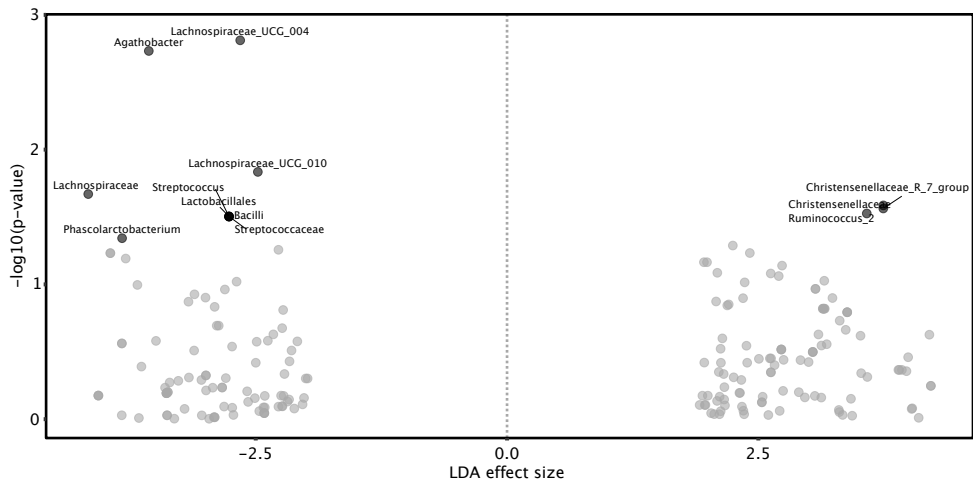

Supplement: jiad604_Supplementary_Data [file jiad604_supplementary_data.zip › figure 2S.pdf]
